# Human Chorionic Villous Differentiation and Placental Development

**DOI:** 10.3390/ijms23148003

**Published:** 2022-07-20

**Authors:** Junya Kojima, Masanori Ono, Naoaki Kuji, Hirotaka Nishi

**Affiliations:** Department of Obstetrics and Gynecology, Tokyo Medical University, Tokyo 160-0023, Japan; kojima_j@tokyo-med.ac.jp (J.K.); naoaki@tokyo-med.ac.jp (N.K.); nishih@tokyo-med.ac.jp (H.N.)

**Keywords:** placenta, iPS cells, fetal growth retardation, hypertensive disorders of pregnancy, pregnancy, gestational diabetes

## Abstract

In humans, the placenta provides the only fetomaternal connection and is essential for establishing a pregnancy as well as fetal well-being. Additionally, it allows maternal physiological adaptation and embryonic immunological acceptance, support, and nutrition. The placenta is derived from extra-embryonic tissues that develop rapidly and dynamically in the first weeks of pregnancy. It is primarily composed of trophoblasts that differentiate into villi, stromal cells, macrophages, and fetal endothelial cells (FEC). Placental differentiation may be closely related to perinatal diseases, including fetal growth retardation (FGR) and hypertensive disorders of pregnancy (HDP), and miscarriage. There are limited findings regarding human chorionic villous differentiation and placental development because conducting in vivo studies is extremely difficult. Placental tissue varies widely among species. Thus, experimental animal findings are difficult to apply to humans. Early villous differentiation is difficult to study due to the small tissue size; however, a detailed analysis can potentially elucidate perinatal disease causes or help develop novel therapies. Artificial induction of early villous differentiation using human embryonic stem (ES) cells/induced pluripotent stem (iPS) cells was attempted, producing normally differentiated villi that can be used for interventional/invasive research. Here, we summarized and correlated early villous differentiation findings and discussed clinical diseases.

## 1. Placental Function

The placenta is an organ limited to the gestational period that is essential for fetal development in the uterus. Primary functions of this organ include (1) exchange of metabolic substances and gas between the mother and fetus; (2) maintenance of pregnancy via hormone production and secretion; (3) protection of the fetus from the maternal immune system (immunological tolerance).

## 2. Villous Differentiation and Placental Formation

### 2.1. From Villi Differentiation to Early Placental Formation

The fertilized egg becomes a blastocyst on the fifth day post-fertilization. This blastocyst consists of an inner cell mass (ICM) and a trophectoderm (TE), the former being the fetal part and the latter being the placenta. In the second embryonic week (4th week of gestation), the blastocyst attached to the endometrium progressively implants into the endometrium, where the TE differentiates into the syncytiotrophoblast (ST) and cytotrophoblast (CT). Around the ninth embryonic day (3 weeks and 2 days of gestation), vacuoles appear in the ST, which eventually fuse to form lacunae. On the other hand, around the 12th embryonic day (3 weeks and 5 days of gestation), capillaries become hyperemic and dilate in the endometrium at the implantation site, forming sinusoids. Then, the ST further invades and erodes the sinusoids, finally leading to a connection between the ST and the sinusoidal endothelial cells where early uteroplacental circulation is formed [1].

After the 13th embryonic day (3 weeks 6 days gestation), the CT invades the ST and forms the primary villi. At the third embryonic week (5th week of gestation), the CT penetrates into the ST and invades the endometrium. Adjacent primary villi also fuse to form a cell layer or the cytotrophoblastic shell, which covers the entire contact surface of the endometrium. Inside the villi, mesodermal cells on the outer wall of the embryo or from the CT invade the central axis to form secondary villi, from which a large number of villous branches ending in the intervillous spaces develop. At the end of the third embryonic week (5th week of gestation), the blood and vascular system differentiate into villous capillaries in the mesodermal villi core. This vasculature eventually connects to the chorionic plate and the capillaries that occur within the connecting stalk, as well as the fetal heart and blood vessels that begin beating at the fourth embryonic week (6th week of gestation), leading to the fetal-placental circulation system. After the fetal placental circulatory system is established, maternal blood and fetal blood are separated from the maternal surface by the intervening cells and tissues, such as the ST-CT-villi interstitial connective tissue, villi capillary endothelium, fetal blood, and maternal blood [2].

### 2.2. Formation of the Trophoblast

Trophoblasts can be broadly divided into two types—the trophoblast at the tip that invades the endometrium and the trophoblast that exists in the villous space due to the infiltration. The former is a trophoblast that infiltrates the endometrium, known as extravillous trophoblast (EVT). EVTs infiltrate deeply from the trophoblastic basement membrane toward the endometrium while forming a columnar cell column that causes remodeling of the uterine spiral artery. EVTs further infiltrate and temporarily close the arterioles; eventually, the vascular endothelial cells are completely replaced by trophoblasts. Furthermore, since the smooth muscle layer of the blood vessel wall is also replaced by trophoblasts, the proximal arteriole of the spinal artery endometrium is constantly in a dilated state, ensuring an abundant blood supply to the intervillous space.

The latter trophoblasts form villi; hence, they are termed villous trophoblast cells. These cells are maintained as a continuous single layer of CT on the basement membrane of the trophoblast. As the villi develop in the cavity, they become responsible for the formation of the ST that is in contact with maternal blood and covers the outermost surface of the villi. The main role of the ST is to facilitate gas and nutrient exchange between the mother and the fetus [3]. ST also secretes several hormones to maintain pregnancy, such as the human chorionic gonadotropin (hCG) and human placental lactogen (hPL). The proportion of CT decreases with the progression of pregnancy, and ST is mainly observed in the placenta at the end of labor.

### 2.3. Placental Blood Vessel Development

At around 20 embryonic days (5 weeks gestation), the basic structure of the villi is completed, which is then followed by placental villi angiogenesis. This angiogenesis is not the invasion of fetal blood vessels into the placenta but the formation of new capillaries. At this point, the villi exist as primary or secondary villi, with mesenchymal cells at the center. Hofbauer cells derived from mesenchymal cells appear when the first blood vessels are formed in the secondary villi, and the expression of angiogenic factors from these cells and the surrounding stromal cells leads to angiogenesis in the placenta. In addition, decidual cells and macrophages on the maternal side also express angiogenic factors.

Around the sixth week of gestation, a vascular basement membrane is formed. By the end of the 13th week of gestation, blood vessels in the villi repeat their formation and branching, with no functional differentiation and numerical increase. Then, myofiber cells develop around the blood vessels around the 15th week of gestation, completing their basic structure. The formation of blood vessels continues until the middle of gestation as the placenta grows. During the third trimester, blood vessel branching does not occur in the villi, and a villus morphology suitable for gas exchange is formed [4,5].

## 3. Placental Formation and Hypoxia

In early pregnancy, blastocysts are exposed to severe hypoxia (2–3% or low PO_2_ of 15–20 mmHg) in the uterus 6 days after conception [6]. From the 10th to 12th weeks of gestation, the trophoblast occludes the spiral artery and prevents the influx of maternal blood into the trophoblastic space. Therefore, the placenta and fetus are placed under hypoxic conditions. In fact, from 8 to 10 weeks of gestation, the oxygen tensions of the placenta and endometrium were 17.9 mmHg and 39.6 mmHg, respectively. It has been reported that the placental oxygen tension at 11 weeks of gestation is about 1/4 of that of the term decidua [6]. From the 12th to 13th week of gestation, the trophoblastic obstruction in the spiral arteries becomes loose, allowing the maternal blood inflow into the trophoblastic space. This makes the placental oxygen tension equivalent to the endometrial oxygen tension [7]. In the early stages of gestation, the placenta and fetus develop under hypoxic conditions. This hypoxic-ischemia-reoxygenation process is normal, physiological, and essential for normal fetal and placental development [8,9]. As mentioned above, hypoxic conditions are not always harmful to cells and can sometimes be beneficial and protective.

Trophoblastic infiltration into the maternal tissue is similar to that of tumor cells, but unlike tumor cells that infiltrate indefinitely and randomly, trophoblastic infiltration occurs within a controlled range [10]. For example, in invasive tumors, a hypoxic environment induces the expression of vascular endothelial growth factor (VEGF) and angiogenesis [11]. It is postulated that this series of reactions is greatly involved in cell migration and infiltration [12]. Among invasive trophoblasts in vivo, only trophoblasts located near the fetal side of villi proliferate [13]. Furthermore, trophoblasts have a lower proliferative ability and higher infiltration ability as they move away from the fetal side of the placental villi. As trophoblasts infiltrate, they become exposed to a high oxygen environment, which halts cell proliferation and causes the continuation of the trophoblast infiltration [14,15]. This phenomenon is controlled spatiotemporally and is extremely important for the continuation of pregnancy [16].

The hypoxic environment in early pregnancy plays an important role in controlling the proliferation and infiltration of these precise trophoblasts. It is speculated that the proliferative nature of trophoblasts under hypoxic conditions may contribute largely to the development of the placenta before fetal development in the early stages of gestation [17,18]. Furthermore, physiological hypoxia during the early stages of pregnancy is thought to enhance the angiogenic activity of the fetal placental endothelium [19]. At 8–9 weeks of gestation, the expression of reactive oxygen species (ROS) scavenging enzymes in the trophoblast cytoplasm and mitochondria is low, making the trophoblasts susceptible to damage by oxidative stress. The expression of the ROS scavenging enzymes increases after 10 weeks of gestation and gradually allows resistance to oxidative stress [20,21,22]. Thus, the relationship between placental formation and oxygen tension is important, and its abnormalities are closely related to the pathogenesis of HDP and FGR.

Various factors influence the characteristic properties of trophoblasts under hypoxic conditions. Among them, hypoxia-inducible factor (HIF)-1 is a typical transcription factor activated by hypoxia. It has a basic helix-loop-helix-PAS (bHLH-PAS) region consisting of HIF-1α and HIF-1β subunits. HIF-1 regulates the transcription of various genes in a cell-specific manner under hypoxic conditions. Under normal oxygen levels, the proline residues of HIF-1 are hydroxylated by proline hydroxylase (PHD), inducing ubiquitination and proteasome degradation by the ubiquitin ligase Von Hippel Lindau (VHL) disease-causing gene product, pVHL. However, under hypoxic conditions, PHD is inhibited, thereby allowing HIF-1 to have transcriptional activity. HIF-1 is also known to induce a transcriptional response to hypoxic stimulation by binding to hypoxia response elements (HRE) present in the promoters and enhancers of genes involved in the glycolytic pathway, sugar transport, and angiogenesis [23,24]. Downstream enzymes of HIF-1, such as the vascular endothelial growth factor (VEGF), glucose transporter-1 (GLUT1), transforming growth factor β3 (TGFβ3), etc., also affect placental formation [25,26,27]. Mice lacking VHL or HIF-1β undergo intrauterine fetal death due to placental dysfunction [28,29]. Furthermore, VHL and HIF-1β are reported to be involved in the differentiation and invasion of human and mouse trophoblasts [30,31].

TGFβ3 inhibits EVT infiltration by inducing the switch from integrin α1 to integrin α5 under hypoxic conditions and promoting the expression of TGFβ3 [32]. TGFβ3 is highly expressed in the placenta from 6 to 9 weeks of gestation and suppresses trophoblastic invasion. Normally, the expression of TGFβ3 decreases around the ninth week of pregnancy. If the expression is increased after 9 weeks, the placenta becomes shallowly infiltrated by trophoblasts and suppresses the expression of angiogenic genes, causing HDP [33,34,35]. TGFβ3 also regulates genes involved in the cell cycle and is involved in FGR morbidity [36]. In addition, the insulin-like growth factor 2 (IGF-II) has also been reported to affect the trophoblastic infiltration under hypoxia [37].

Ten-eleven translocation 1 (TET 1), an important gene that has a role in DNA demethylation, affects trophoblast infiltration [37,38]. Thus far, various experimental systems have reported that trophoblasts proliferate under hypoxic conditions. Trophoblasts, which proliferate actively in the body from 6 to 10 weeks of gestation, have decreased mitotic figures from 10 to 12 weeks of gestation when exposed to maternal blood [39]. A study has revealed that under hypoxic conditions, the HTR-8 SVneo cell line of the early human trophoblast has increased proliferative capacity and decreased infiltration capacity into the Matrigel [40]. Trophoblasts isolated from living organisms also had an enhanced proliferative capacity under 2% oxygen concentration compared to those under 20% oxygen concentration [41]. There were also reports of increased VEGF expression mediated by the renin–angiotensin system and increased infiltration ability mediated by the notch signaling under hypoxic conditions [42,43]. On the other hand, recent studies revealed that HIF2α is involved in the placental formation and that HIF2α in the decidua allows the infiltration of the chorion in a mouse model [44,45]. In any case, the relationship between hypoxic conditions and the proliferation and infiltration of trophoblasts is important, and any abnormalities in these processes may be closely related to the pathogenesis of HDP and FGR.

The differentiation between CT and polynuclear ST is performed in a microenvironment with a relatively high oxygen tension. In a study wherein human villous membrane cells were cultured in vitro under different O_2_ levels, an atmospheric (21%) O_2_ level promoted natural CTB cell fusion to STB, while low O_2_ levels (<11%) markedly reduced cell fusion. Low O_2_ levels were found to downregulate the hormone levels secreted by STB [31,46]. The inhibition of STB differentiation under hypoxic conditions during the first phase of pregnancy is partially dependent on the intact HIF complex. In HIF-mutant mice, CTB differentiates exclusively into STB, suggesting that in the absence of hypoxic conditions or stimulation, CTB, by default, differentiates into STB [47]. HIF-1β deficiency (ARNT) in primary CTB can restore the secretion of STB-producing HCG [31]. Additionally, the expression of trophoblast-specific HIF-1α, which mimics prolonged hypoxia, causes vascular development such as decreased branch morphogenesis, changes in the mesenteric space, and impaired spiral arterial remodeling [48].

## 4. Formation of the Decidua

Embryo implantation into the endometrium is only possible during the implantation phase, and the sex steroid hormones secreted from the ovary are important for the regulation of endometrial differentiation. The endometrium responds to sex steroid hormones and factors from the embryo, allowing for endometrial changes suitable for embryo implantation. On the 7.5th day post-ovulation, the human embryo is already buried under the epithelium of the endometrium, and the trophoblast cells on the uterine side are enlarged and activated. On the 12th day post-ovulation, lacunar spaces are formed in the trophoblast cell layer, and communication with the maternal blood flow is started. The human chorionic gonadotropin (hCG), which is produced in large quantities by trophoblasts and shares a receptor with LH, reaches the corpus luteum via the maternal bloodstream, which then stimulates progesterone production and maintains embryo implantation.

Immediately after implantation of the blastocyst, the endometrium becomes edematous due to the action of progesterone, the uterine spiral arteries also develop remarkably, and the endometrial stromal cells undergo decidualization. In addition to the mechanism of embryo–maternal implantation induction via the endocrine system, the immune system also plays an important role. In addition, the mother’s immune system develops immune tolerance to the fetus due to the antigen derived from the paternal line and accepts the fetus. One of the immune mechanisms is the proliferation of regulatory T (Treg) cells that specifically recognize the father-derived antigen expressed by the fetus in the mother’s body and suppress the immune response against the fetus [49].

Furthermore, it is thought that normal placental formation is established by the active recognition of fetal-derived trophoblasts by the maternal immune system even during the placental formation stage after implantation. The human placenta is a hemochorial placenta where trophoblasts infiltrate the decidua deeply, allowing for wide contact of fetal and maternal tissues. Immediately after blastocyst implantation, when trophoblasts infiltrate the endometrium, the endometrium becomes the decidua. The number of immune cells is increased at the implantation site in the decidua; the majority are decidual natural killer (dNK) cells, macrophages, and dendritic cells (APCs). It has also become clear that dNK cells and APCs are involved in trophoblast infiltration and placental angiogenesis. From here on, we will outline the role of various immunocompetent cells in the endometrium.

### 4.1. Natural Killer (NK) Cells

#### 4.1.1. Decidual Natural Killer (dNK) Cells

Initially, NK cells were reported as lymphocytes that were cytotoxic to tumor cells. Later, it was reported that NK cells have both cytotoxicity and cytokine-producing ability in a lymphocyte population separate from T cells and B cells [50]. In humans, NK cells are recognized as CD56+/CD3− cells. There are two populations of NK cells in the blood-cytotoxic NK cells (CD56dim/CD16+) and cytokine-producing NK cells (CD56++/CD16−), with the latter accounting for about 90% of the cell population [51].

The dNK cells in the human endometrium are CD56++/CD16−. However, dNK cells have many granules in the cells and have different properties from CD56++/CD16− NK cells in the blood [52]. Even during non-pregnancy, dNK cells are present in the endometrium; a small number are present in the proliferative phase and early secretory phase, and the number of cells increases in the late secretory phase. In early pregnancy, the number of dNK cells further increases, accounting for about 70% of the total leukocyte count in the decidua during the first trimester and are localized near trophoblasts [53]. Interestingly, dNK cells also increase during delivery [54,55,56]. After the second trimester, dNK cells decrease [54,55]. dNK cells were reported to originate from the following: (1) peripheral blood CD56++/CD16− NK cells, (2) peripheral blood CD56dimCD16+ NK cells, (3) immature NK progenitor cells in the uterus, and (4) hematopoietic stem cells [57,58,59]. In addition, the CXCL12/CXCR4 signaling pathway is used for dNK cell migration to the implantation site [60,61].

#### 4.1.2. Role of dNK Cells

dNK cells regulate the degree of trophoblast invasion into the decidua [62]. dNK cells express HLA receptors, killer cell immunoglobulin-like receptors (KIRs), CD94/NKG2A, and ILT2. These are HLA-C, HLA-E, and HLA-G receptors expressed in trophoblasts, respectively, and contribute to the immunological tolerance of trophoblasts at the fetomaternal interface. dNK cells are also involved in decidua-helicine artery remodeling, increasing the blood flow to the placenta and playing an important process in fetal development [63,64]. In animal models, NK cell-deficient mice were reported to have a reduced blood flow to the placenta [65].

### 4.2. Decidual Macrophages

Monocytes in the circulating blood are precursors of decidual macrophages. During pregnancy, the phagocytic activity of monocytes decreases, protecting the alloantigen fetus. Decidual macrophages are mainly located in the interstitial site just below the endometrial epithelium and are localized around the fetomaternal interface during the implantation period. Decidual macrophages play a role in transmitting immune information derived from embryonic paternity to the mother and in tissue remodeling of the implantation site.

#### 4.2.1. M1 and M2 Macrophages

Macrophages can be classified into two types—those activated by the classical pathway (M1) and those activated by the alternative pathway (M2) [66]. M1 and M2 have different patterns of surface markers and cytokine secretion [67]. M1 macrophages are associated with inflammation, have a high antigen-presenting ability, secrete a large number of cytokines, including IL-12, IL-23, and ROS, and are involved in the Th1-type response [68]. M2 macrophages secrete cytokines such as IL-4, IL-10, and VEGF, which are involved in tissue remodeling and immunosuppression by promoting Th2-type immune responses. Studies on transcription factors that control macrophage differentiation have indicated that STAT1, C/EBP-α, C/EBP-δ, and NF-κB are involved in M1 macrophage differentiation, while PPARs, STAT3, STAT6, and C/EBP-β are involved in M2 macrophage differentiation [69]. The inflammatory environment in preeclampsia (HDP) increases M1-type digital macrophages [70].

#### 4.2.2. Phenotypes of Decidual Macrophages

M1 macrophages express CD80 and CD86 as surface markers, while M2 macrophages express CD206, CD209, and CD163. After the implantation period during pregnancy, lymphocytes and polymorphonuclear leukocytes do not increase at the implantation site, whereas decidual macrophages increase before delivery. After the complete formation of the placenta, decidual macrophages differentiate mainly to M2 to prevent fetal rejection and allow fetal growth until delivery. Recently, M2 expressing Tim-3 has been attracting attention as an important biomarker for maintaining immune tolerance [71]. Furthermore, decidual macrophages in the first trimester are classified by the expression of CD11c. CD11c-high decidual macrophages had lower expression of phagocytic receptors (CD209 and CD206) than CD11c-low decidual macrophages. CD11c-high decidual macrophages function in lipid metabolism, inflammation, antigen processing, and immunoregulation, while CD11c-low decidual macrophages express genes involved in growth and development regulation and extracellular communication [72].

### 4.3. Regulatory T (Treg) Cells

Treg cells have the function of suppressing the excessive immune response that underlies autoimmune, inflammatory, and allergic diseases. On the other hand, Treg overactivation may suppress the immune response to cancer and promote its progression. The transcription factor Foxp3 is involved in the regulation of Treg cell development and differentiation. Mutations in the Foxp3 gene were identified as the cause of IPEX (immune dysregulation, polyendocrinopathy, enteropathy, X-linked syndrome), which is a human autoimmune disease also seen in scurfy mice. Immature T cells in the thymus express Foxp3 upon presentation of a self-antigen by thymic epithelial cells and induce differentiation into Treg [73,74].

#### Role of Treg Cells

Immune tolerance exists during pregnancy when the fetus is accepted. For example, when a Balb/c female mouse mates with a C57BL/6 male mouse and becomes pregnant, tumor cells derived from C57BL/6 engraft in the pregnant Balb/c female mouse. After delivery, tumor cells derived from C57BL/6 are rejected [75]. This indicates that immune tolerance is achieved in female mice to paternal antigens during pregnancy, which disappears after delivery. Treg cells play an important role in immune tolerance during pregnancy [76]. Decreasing Treg cells during implantation results in implantation failure in allogeneic pregnancies but has no effect in syngeneic pregnancies. In other words, when the number of Treg cells decreases at the time of implantation, embryos expressing an allogeneic antigen are rejected. The expression of Foxp3, a Treg cell marker, is decreased in the endometrium during implantation in unexplained human infertility cases [77]. When Treg cells decrease in the early stages of pregnancy, miscarriages of both male and female fetuses occur in allogeneic pregnancies, but only of male fetuses in syngeneic pregnancies [78,79]. Treg reduction induces an immune tolerance not only to the major histocompatibility complex (MHC) but also to minor histocompatibility antigens (MiHA) such as the male-specific antigen, SRY. These results show that Tregs function to maintain pregnancy.

Th1/Th2/Th17/Treg cell imbalance and abnormal Treg ratios have been reported as the mechanisms involved in implantation failure. In patients with recurrent miscarriages, the ratios of Th1 cells to Th2 cells and of Th17/Treg cells are high, predominantly having Th1 and Th17 cells, respectively [80,81]. There is a close interaction between Treg proliferation and IL-17 secretion. When IL-17 binds to the IL-17 receptor, Treg cells proliferate. Conversely, Treg cells suppress Th17 cell proliferation and IL-17 secretion via Il-10/TGF-β [82]. Administration of IL-17 to mice markedly increased the abortion rate; however, administration of Treg cells to mice significantly increased IL-10 and TGF-β expression and prevented abortion [83]. In addition, a report examining postpartum decidua showed that Treg cell expression was elevated in placenta accreta compared to that in the normal placenta, suggesting that Treg cells may be involved in EVT infiltration [84].

## 5. Genes Involved in Villous Differentiation

**Figure 1:**
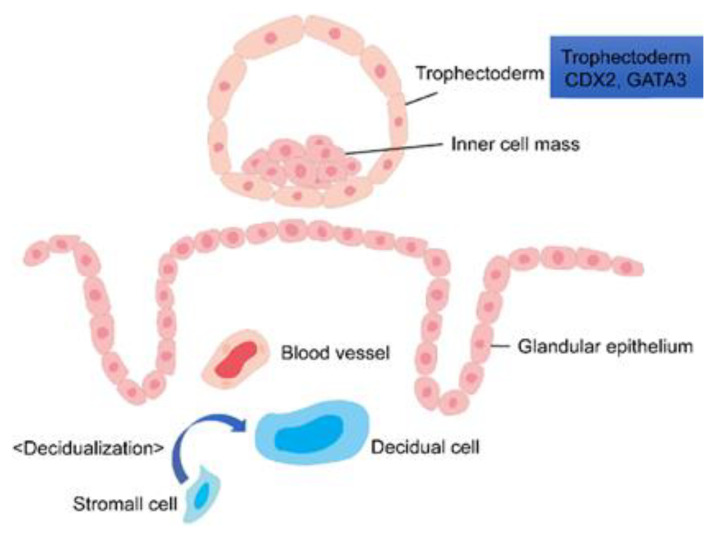
Placental development immediately before implantation and involved genes in the trophectoderm.

**Figure 2:**
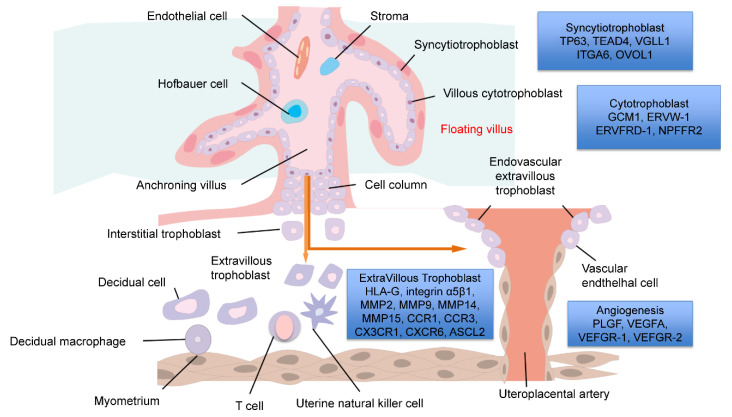
The fetomaternal interface. The major trophoblast subtypes and maternal cells are illustrated. Genes involved in the formation of the cytotrophoblast, syncytiotrophoblast, extravillous trophoblast, and angiogenesis are described.

Studies have been conducted to understand villous differentiation using early abortion samples and transgenic mice. In recent years, new experimental tools have become available, such as blastocyst analysis and the above-mentioned induction of trophoblast differentiation using ES cells and iPS cells. Here we describe some known typical genes that contribute to placental differentiation (Figure 1 and Figure 2 and Table 1).

## 6. Diseases Involving Placental Dysfunction

### 6.1. Fetal Growth Restriction (FGR)

Scattered infarcts are characteristic placental histological findings in FGR. However, images of placental infarcts are not FGR-specific and can only be seen in approximately 25% of the FGR placenta. In addition, nodules in ST, CT thickening, fibrinoid deposition, decreased vascular bed, decreased volume of villi, decreased villous space, and non-specific inflammation were observed, which all decreased the blood flow in the fetus-placenta-uterus [118]. Biologically, VEGF-A expression is elevated in FGR placentas. This indicates that a decrease in fetal-placental-uterine blood flow during placental formation induces the expression of angiogenic factors [119]. Furthermore, in FGR, the proportion of uNK cells in the decidua is reduced, as well as the expression of VEGF-A, PGF, interleukin-10, and other angiogenic factors [120,121,122]. In FGR, the expression of HLA-G in EVT is reduced, resulting in the disruption of the fetomaternal immune tolerance and the occurrence of non-specific inflammatory findings. In the placenta of FGR, the mTORC1 activity of Mechanical TOR (mTOR), a serine-threonine kinase protein that regulates cell survival, metabolism, growth, and proliferation, is inhibited [123]. The mTOR pathway regulates the expression and activity of placental transporters responsible for the transport of amino acids, fatty acids, and glucose. Many of these transporters are underexpressed in FGR, resulting in functional loss of the placenta [124,125,126]. Thus, it has been suggested that placental hypoplasia is one of the causes of FGR.

### 6.2. Hypertensive Disorders of Pregnancy (HDP)

Placental features of preeclampsia include fibrinoid deposition on the vessel wall, macrophage hyperplasia, villus hypoplasia (especially decreased villus count on the maternal surface, decreased villus diameter, and decreased vascular bed), and ST nodules [127,128]. The two-step theory is a well-known cause of preeclampsia [129], which states that a hypoxic state due to vascular remodeling failure secondary to the infiltration of extravillous trophoblasts into the spiral artery results in a decreased blood flow to the villous lumen and an insufficient oxygen supply [130]. Under hypoxic conditions, trophoblasts produce soluble endoglin (sEng) and soluble fms-like tyrosine kinase-1 (sFlt1). sEng constricts blood vessels and restricts blood flow. In addition, sEng and sFlt1 inhibit VEGF and PGF receptors, resulting in the inhibition of angiogenesis and proliferation of vascular endothelial cells. sEng and sFlt1 are expressed transplacentally and are also found in the maternal circulation, inducing maternal vascular endothelial damage along with placental hypoplasia [131,132]. In addition, the heat shock protein 70 (HSP70) released from the placenta, high mobility group box 1, and tissue factor (TF), which promotes coagulation, also contribute to the pathogenesis of HDP [128].

The reason for the occurrence of helical artery hypoplasia has not yet been elucidated, but the interaction of the maternal immune system and the fetal (paternal) major histocompatibility complex (MHC) antigen causes a local fetomaternal immune response. It is believed that promoting tolerance is essential for normal placental formation, and any hindrance to immune tolerance is thought to lead to the development of HDP [133]. Treg cell dysregulation is also involved in the disease. The number of Treg cells and the ratio of Treg/Th17 cells are significantly reduced in preeclampsia [134]. In HDP, the expression of Foxp3, a Treg-specific transcription factor, is decreased, and the expression of the retinoic acid receptor-related orphan nuclear receptor γt (RORγt), a Th17-specific transcription factor, is increased compared to those in healthy pregnant women [135]. As described above, it is suggested that in HDP, the conversion from Treg cells to Th17 cells occurs, resulting in an abnormal immune state in which inflammation is induced. In addition, maternal factors such as obesity, hypertension, and autoimmune diseases increase the risk of developing HDP. Furthermore, the degree of inflammation of maternal blood vessels before pregnancy may contribute to the onset of HDP [136,137,138]. In recent years, it has been reported that autophagy is involved in placental hypoxia and that autophagy failure is associated with the onset of PE [139,140].

### 6.3. Gestational Diabetes Mellitus (GDM)

The histological findings of the placentae of GDM patients have been reported to include a significant increase in fibrinoid necrosis, infarct image, and immaturity of villi compared to those of the normoglycemic control group. It is also reported that the fetal/placental weight ratio was significantly lower in the diabetic group [141,142]. GDM placentae have increased leptin concentration, increased expression of the TNF-α signaling gene, and increased IL-1 and IL-8 receptor genes compared to normal placentae [143,144,145]. These inflammatory cytokines are speculated to be involved in maternal insulin resistance. In addition to the inflammatory reaction, increased placental oxidative stress and ER stress contribute to the onset of GDM [146,147,148]. In the placenta, glucose is taken up via the glucose transporter (GLUT), and the fetus obtains glucose from the maternal circulation via the placenta. Furthermore, GLUT-1 is expressed in ST and is involved in glucose uptake from the maternal circulatory system. GLUT-1 is upregulated in the placentae of GDM patients, in addition to the upregulated expression of GLUT-3 and GLUT-4 in other organs [149,150].

## 7. Trophoblast Research Tool

The placenta is involved in the development of various diseases. However, it is difficult to investigate the causes of disease in the human placenta. Although it is possible to obtain the placenta of the diseased patients, it is technically and ethically difficult to reproduce the findings obtained from the placenta in vivo. Various common in vitro models have been used so far. For example, a primary culture using placental tissue after delivery was performed; however, a complete trophoblast was difficult to isolate from the placental tissue, and it was difficult to maintain the culture for a long period, thus making it difficult to use for research. In recent years, single-cell analysis has become possible, bringing new findings to placental research [151,152].

Choriocarcinoma cell lines such as BeWo, JAG-3, and JAR have the advantage of being easily cultured, although their origins are different from those of normal villi [153,154,155]. Choriocarcinoma cell lines are difficult to differentiate from CT to EVT in culture but are easily differentiated from the ST lineage. Therefore, placenta-derived EVT immortalized cell lines such as HTR 8/SVneo and Swan71 are used instead. However, in reality, the phenotype is slightly different from that of the living placenta [156,157]. It is difficult to evaluate any of these differentiation culture systems in terms of whether or not they reflect the state in vivo. In addition, it is difficult to reproduce the research in primary cultures because of the effect of the patient genetic background complicating the culture system. In order to overcome these problems, culture systems using ESCs and iPSCs are established to differentiate into trophoblasts [46,158,159]. We are currently studying this culture system [160]. We focused on KRT7, which is a general surface marker of trophoblasts. We treated iPSCs with BMP4 and reported the results of a comprehensive analysis of KRT7-positive cells [3,161].

In recent years, long-term culture systems of the human CT (monolayer and spheroids) and organoids are produced from the placenta in the early stages of pregnancy without using ESCs or iPSCs [162,163]. There is also a report of a culture system treated with naive iPSCs that was used for research on the differentiation of fertilized eggs into a trophectoderm system [164]. These stem cell and CT culture systems, and organoid production, may be advantageously used to study the process of differentiation from CT to ST and EVT to some extent. However, it is impossible to confirm whether the situation in vivo is reproduced.

## 8. Conclusions

We have described the morphological development of the placenta and villi and their differentiation from the viewpoint of molecular biological markers, taking into account their relationship with pregnancy-related diseases. The early placental formation is difficult to study from an ethical point of view, and there are many unclear points about its pathophysiology. However, recent studies have reported the development of various culture systems for understanding early placental formation. The issue is how to utilize these new culture systems, which have become feasible in recent years, for investigating the causes of various diseases related to the placenta and developing treatment strategies.

## Figures and Tables

**Table 1:** Summary of each gene associated with placentation.

Human First-Trimester Trophoblast Stage and Vascular Development	Gene Symbol	Gene Description
Trophectoderm (TE)	Caudal Type Homeobox 2 (CDX2)	The interaction of Oct4 and CDX2, which are typical undifferentiated markers that affect the inner cell mass, leads to the differentiation of the trophoblast and the inner cell mass. Although CDX2 is known to be expressed in the trophoblast, the detailed mechanism of trophoblast differentiation is unknown.
	GATA Binding Protein 3 (GATA3)	GATA3, a transcription factor expressed in trophoblasts, is involved in the differentiation of trophoblasts into villi as well as the infiltration and migration of the villi into the maternal surface. It is thought to play an important role in the placental formation [85,86].
Cytotrophoblast (CT)	Tumor Protein P63 (TP63)	TP63 is a member of the P53 tumor suppressor family. Through the control of epithelial-mesenchymal transition, cell adhesion, and matrix degradation pathways, TP63 suppresses CT differentiation into EVT and maintains a proliferative CT state [87]
	TEA Domain Transcription Factor 4 (TEAD4)	Transcription factors of the TEAD family are the ultimate intranuclear effectors of the Hippo pathway. Among these, TEAD4 regulates cell growth, proliferation, and homeostasis in CT [88,89,90].
	Vestigial-Like Family Member 1 (VGLL1)	VGLL1 is a co-transcriptional activator of TEAD4; when VGLL1 expression decreases, the expression of TP63, which is a marker of CT, also decreases. For this reason, VGLL1 is thought to be involved in the maintenance of CT [91,92].
	Integrin Subunit Alpha 6 (ITGA6)	ITGA6 is a cell surface protein that constitutes the major adhesive receptor for laminin. Abundant laminin is present in the stem cell niche, and ITGA6 is involved in cell proliferation and self-renewal [93].
	Ovo-like Transcriptional Repressor 1 (OVOL1)	OVOL1 regulates TP63 expression. OVOL1 is necessary to suppress the differentiation of CT into ST and maintain its state by inhibiting the expression of syncytin1 and syncytin 2 [94].
Syncytiotrophoblast (ST)	Glial Cell Missing Transcription Factor 1 (GCM1)	GCM1 is a gene that inhibits the differentiation from CT to EVT and induces differentiation into ST by fusing cells. GCM1 is controlled by GATA3 [95,96].
	Neuropeptide FF-Amide Peptide Precursor (NPFF)	The role of neuropeptide FF (NPFF) is well known in the central nervous system. NPFF receptor 2 (NPFFR2) mRNA is abundant in the placenta; however, the function of NPFF-NPFFR2 in placental development is unknown. NPFF acts via NPFFR2, promotes the expression of syncytin 1 and 2 via GCM1, and is involved in ST formation [97].
	Endogenous Retrovirus Group W Envelope Member 1, Envelope (ERVW-1)	ERVW-1, a gene encoding the syncytin-1 protein, is derived from an endogenous retrovirus. Syncytin-1 promotes cell fusion. Genes derived from retroviruses play an indispensable role in placental formation. The expression of ERVW-1 is regulated by GCM-1 [98,99].
	Endogenous Retrovirus Group FRD Member 1, Envelope (ERVFRD-1)	ERVFRD-1 is a gene encoding the syncytin-2 protein. Like ERVW-1, ERVFRD-1 is also derived from an endogenous retrovirus. Syncytin-2 also promotes cell fusion and is also controlled by GCM1 [100,101]
Extravillous trophoblast (EVT)	Major Histocompatibility Complex, Class I, G (HLA-G)	HLA-G is the most representative gene expressed in EVT. It is classified as a human leukocyte antigen, which is a human major histocompatibility complex. HLA-G contributes to immunosuppression to establish pregnancy and allow the fetus to escape maternal immunity [102].
	Integrin	Integrin is a protein on the cell surface and is a cell adhesion molecule. It is a heterodimer consisting of two subunits, the α and β chains. Integrin α5β1 is expressed in EVT [103]. Pregnancy-specific glycoproteins (PSGs) are secretory proteins present in the maternal placenta. There are 11 PSG genes in humans, and PSG1 interacts directly with integrin α5β1 [104].
	Matrix Metalloproteinase (MMP)	The MMP family currently has 28 members (MMP 1 to 28), and the expression of MMP2, MMP9, MMP14, and MMP15 has been reported in EVT. MMPs degrade extracellular matrices and proteins expressed on the cell surface [105,106,107,108].
	Chemokines and Chemokine Receptors (CCR)	Chemokines are small-molecule polypeptides involved in cell proliferation, differentiation, apoptosis, angiogenesis, hematopoiesis, tumor promotion, and inflammatory diseases (85,86). Chemokines play an important role in placental function and play a major role in the infiltration of EVT into the maternal decidua, as the chemokine receptors CCR1, CCR3, CX3CR1, and CXCR6 are localized in EVT [109,110,111,112].
	Achaete-Scute Family BHLH Transcription Factor 2 (ASCL2)	ASCL2 is a member of the basic helix-loop-helix (BHLH) family of transcription factors. Its expression is observed in EVT in early pregnancy. While ASCL2 has been reported to be involved in tumor infiltration in breast cancer, it also plays an important role in human EVT formation [31,113].
Angiogenesis	Placental Growth Factor (PGF)	PGF belongs to the vascular endothelial growth factor (VEGF) family. PGF is expressed in vascular endothelial cells in the placenta and plays a role in vasodilation and angiogenesis [114,115].
	Vascular Endothelial Growth Facto A (VEGFA), Fms Related Receptor Tyrosine Kinase 1 (FLT1), and the Kinase Insert Domain Receptor (KDR)	VEGFA, a vascular endothelial growth factor, belongs to the VEGF family and is expressed in trophoblasts and vascular endothelial cells, and is also involved in angiogenesis in the early placenta. VEGFR-1/Flt-1 and VEGFR-2/KDR/Flk-1 have been identified in placental tissues as receptors for the VEGF family [116,117].

## Data Availability

Not applicable.
